# A Historical Perspective on Surgical Manipulation of the Membranous Labyrinth for Treatment of Meniere's Disease

**DOI:** 10.3389/fneur.2021.794741

**Published:** 2021-12-23

**Authors:** Calvin J. Kersbergen, Bryan K. Ward

**Affiliations:** Department of Otolaryngology-Head and Neck Surgery, Johns Hopkins University, Baltimore, MD, United States

**Keywords:** Meniere's disease, hydrops, membranous labyrinth, endolymph, cochleosacculotomy, vertigo, hearing loss

## Abstract

Meniere's disease is an inner ear disorder without a known cause. Endolymphatic hydrops is a swelling of the endolymph spaces that has been observed consistently on post-mortem histology in patients with a history of Meniere's disease but can occur in asymptomatic individuals and in association with other diseases. Since its discovery, Meniere's disease has been a disorder managed primarily by otolaryngologists. Surgical treatments, therefore, have accompanied attempts at medical management. Inspired by patients' sensations of ear fullness and later by the histologic findings of hydrops, surgeons began manipulating the membranous labyrinth to relieve episodes of vertigo while attempting to preserve hearing. This review highlights this history of manipulation of the membranous labyrinth. These procedures indicate a rich history of innovation that parallels developments in otologic surgery. The studies involving patients are uniformly retrospective, with some procedures performed first in animal models of endolymphatic hydrops. Many approaches were endorsed by eminent otologic surgeons. Surgeries on the endolymphatic sac are performed by some surgeons today; however, procedures on the membranous labyrinth resulted in similar symptomatic relief through a minimally invasive technique, in many cases performed using only local anesthetic. Episodic vertigo in patients with Meniere's disease is a distressing symptom, yet spontaneous remissions are common. The reports of procedures on the membranous labyrinth reviewed here consistently indicated fewer vertigo episodes. Variable degrees of hearing loss were common following these procedures, and many were abandoned. Additional innovative surgeries are inevitable, but we must understand better the relationships among endolymphatic hydrops, Meniere's disease pathophysiology, and patient symptoms.

## Introduction

Meniere's disease is a disorder of the inner ear consisting of intermittent, spontaneous episodes of vertigo in combination with other fluctuating ear symptoms including low-frequency sensorineural hearing loss, aural fullness, and tinnitus. The condition is associated with Prosper Meniere who in 1861 identified the inner ear labyrinth as a likely source for symptoms of a syndrome involving episodic vertigo and hearing loss (1). Meniere's disease has a complex and highly variable natural course in which spontaneous remission of vestibular symptoms is common and there is slow progression to end-organ damage.

The etiology of Meniere's disease remains unclear. Histopathologic investigation identified a swelling of the membranous labyrinth called endolymphatic hydrops in temporal bone specimens from patients with Meniere's disease (2, 3), leading to the hypothesis that increased fluid volume within the membranous labyrinth triggers the symptoms of vertigo, hearing loss, tinnitus, and aural fullness. The presence of greater endolymph volume has often been interpreted as an imbalance in fluid homeostasis, resulting from either abnormally increased generation of endolymph or impaired absorption. Excess endolymph might lead to mechanical impairment of sensory transduction, or to ruptures of Reissner's membrane or other areas of the membranous labyrinth, subsequently mixing endolymph and perilymph. The severity of hydrops correlates with the duration of disease and severity of both hearing loss and vestibular symptoms (4, 5). Endolymphatic hydrops, however, is observed also in asymptomatic individuals and in individuals with many different disorders, suggesting that while hydrops is associated with Meniere's disease, it may not cause the disorder (5–9).

Since the etiology remains unknown, targeted medical therapies for Meniere's disease are not possible. Treating physicians address symptoms and often suggest lifestyle modifications in attempts to prevent recurrent episodes of vertigo. Today, physicians often prescribe oral diuretics and betahistine, followed by offering steroid perfusion of the middle ear for patients with recurrent episodes of vertigo. If these treatments fail to control the episodic vertigo, otolaryngologists often consider ablative procedures of the inner ear, invoking the logic that the brain can adapt better to constant asymmetry in vestibular neural activity than to fluctuating activity (10).

Meniere's disease is a disorder primarily managed by surgeons (i.e., otolaryngologists) and since its discovery surgical innovation has accompanied attempts at medical management. In the 20th century, pioneering surgeons, inspired by the observations of endolymphatic hydrops as well as patient descriptions of “fullness” and “pressure” within the ear, developed conservative surgical approaches for those patients with persistent, disabling symptoms despite medical therapy. There were two goals for these procedures: (1) preserve hearing function, and (2) improve homeostasis between endolymph and perilymph by reducing the excess volume of endolymph. These novel procedures included decompression or microsurgical manipulation of the endolymphatic sac and membranous labyrinth. This narrative review will encompass several procedures on the membranous labyrinth, comparing historical success rates for the control of vertigo episodes, and providing perspectives on the historical influences of these procedures and the future of surgery on the membranous labyrinth for treatment of Meniere's disease and other conditions of the inner ear.

## Early Approaches to Manipulate Endolymph in Meniere'S Disease

In 1871 Knapp comprehensively synthesized the symptoms of patients like those described by Meniere, drawing analogies from Meniere's disease to ocular glaucoma and suggesting surgical relief of pressure could be helpful (11). As early as the 1890s Burnett reasoned that removing the ossicles and tympanic membrane could decompress the labyrinth and provide relief (12, 13). The hearing loss caused by these procedures prompted surgeons to consider new targets to preserve hearing while decreasing the theorized high pressure of the inner ear. The lateral semicircular canal was accessible via the mastoid. Jenkins first decompressed the bony lateral semicircular canal in a patient with active Meniere's disease in 1911, reportedly preserving hearing and vestibular function (14). Similar attempts by Lake to decompress the vestibule, however, often resulted in hearing loss and injury to the facial nerve (15). In the 1920s Portmann, also drawing parallels between Meniere's disease and glaucoma, emphasized decompressing the endolymphatic sac (16). His technique was later expanded by William House and others to inserting materials into the endolymphatic sac to shunt endolymph to the subarachnoid space or mastoid (17). These early surgical attempts to decompress the inner ear gained support by the discovery in 1938 that patients with Meniere's disease had the histologic finding of endolymphatic hydrops. Endolymphatic sac procedures remain in use today by some surgeons, with a success rate for Class A/B vertigo control of 70–80% in retrospective studies across different procedural approaches (18), with a high degree of hearing preservation. Unexpectedly, however, in contrast with both shunting procedures in humans and animal models of hydrops generation (19), procedures that block the endolymphatic duct using titanium clips may also achieve vertigo control in patients with Meniere's disease (20). These contradictory approaches, yet similar clinical observations emphasize our incomplete understanding of physiological endolymph circulation and the role of hydrops in disease (21).

## Introduction of the Surgical Microscope and Lempert'S Fenestration Procedure

**Figure 1:**
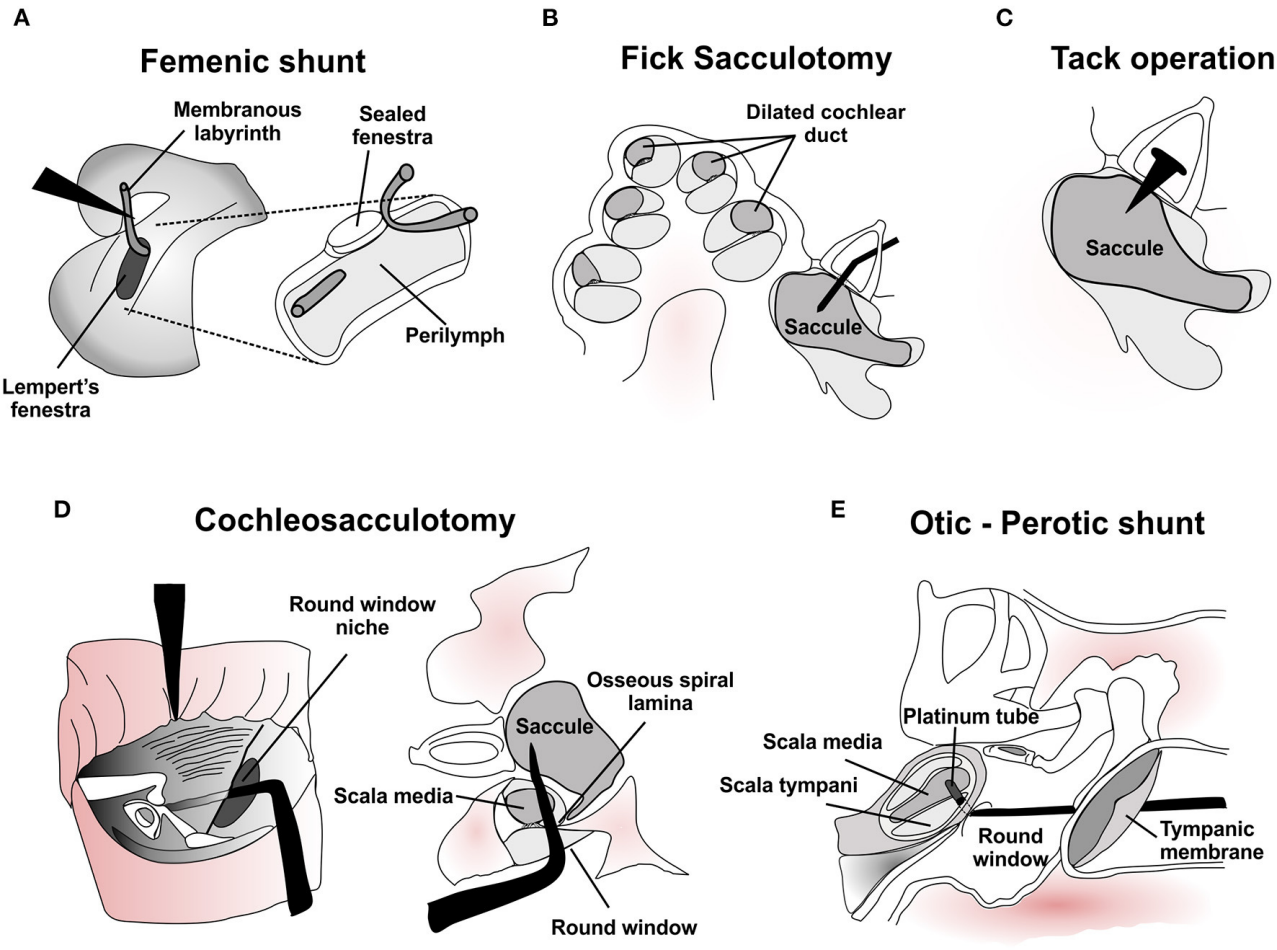
Surgical manipulations of the membranous labyrinth for treatment of Meniere's disease. **(A)** Depiction of Femenic's shunt in the lateral semicircular canal to relieve endolymphatic hydrops and vertigo episodes. Adapted with permission from Femenic (25). **(B)** Histological representation of endolymphatic hydrops in the cochlear duct and saccule, with a Fick sacculotomy needle placed through a fenestration in the stapes to drain excess endolymph. **(C)** Permanent tack placement in the stapes bone was intended to enable repeated decompression of the hydropic saccule in the Cody tack operation. **(D)** Two views of the cochleosacculotomy procedure, where a 90-degree pick is driven through the round window to rupture the cochlear duct and saccule and create a permanent fistula in the osseous spiral lamina. Adapted with permission from Schuknecht (26) and Kinney et al. (27). **(E)** Depiction of the Otic-Perotic shunt procedure, where a platinum tube is placed in the basilar membrane of the basal cochlea to enable decompression of the scala media. Adapted with permission from Pulec (28, 29).

New surgical approaches that manipulated the membranous labyrinth emerged following the adoption by otologic surgeons of the operating microscope and the increased popularity of Lempert's single-stage lateral semicircular canal fenestration procedure for otosclerosis. In 1943 Day attempted to decompress the hydropic membranous labyrinth by inserting a metallic pick through a lateral canal fenestration and into the vestibule. He then cauterized the tissue by running electrical current through the pick (22). Lindsay repeated this approach without cauterization, and reported that patients no longer experienced episodes of vertigo, but uniformly developed hearing loss and sound-induced vertigo (i.e., Tullio phenomenon). He and Cawthorne described the Tullio phenomenon that developed in these cases as having been caused by the presence of a “third mobile window” in the inner ear, later associated with the pathophysiology of superior semicircular canal dehiscence syndrome (23, 24). In 1952 Femenic used Lempert's fenestration of the lateral canal to create a permanent drainage of endolymph into the perilymphatic space. Femenic accessed and divided the membranous labyrinth through the fenestration, and then routed the anterior end outside the labyrinth, leaving the posterior end free to communicate with the perilymph (Figure 1A). He reports all 4 cases having ‘satisfactory' symptom control and preserved cochlear function (Table 1) (25).

**Table 1:** Historical and present outcomes of surgical procedures on the membranous labyrinth for control of vertigo and other symptoms in Meniere's disease.

**Procedure**	**Patient population**	**Follow-up duration**	**Vertigo improvement**	**Aural fullness** **improvement**	**Tinnitus improvement**	**Hearing function**	**Surgical complications**	**References**
Femenic shunt	NR	NR	4/4 (100%)	NR	4/4 (100%)	Air conduction >30 dB HL, bone conduction 10 dB HL with intact ossicular chain.	NR	Femenic (25)
Fick Sacculotomy	No prior improvement with medical treatments. Symptoms of vertigo, HL, tinnitus, aural fullness.	> 1 year for 90% of cases. 19/79 (24%) lost to follow-up.	55/60 (92%)	49/51 (96%)	49/59 (83%)	39/60 (65%) improved hearing, 3/60 (5%) HL.	Mild vertigo and unsteadiness, immediate hearing deterioration before improvement. 1/60 (1.7%) developed labyrinthitis.	Fick (30, 31)
Fick Sacculotomy	4/4 (100%) had > 65 dB HL, >3 years vertigo.	1-5 months.	4/4 (100%)	NR	1/2 (50%)	4/4 (100%) complete HL.	No postoperative complications.	Proud (32)
Fick Sacculotomy	NR	NR	6/6 (100%)	NR	NR	6/6 (100%) severe HL 10-12 h post-op.	NR	Arslan (33)
Fick Sacculotomy	NR	NR	2/5 (40%)	NR	NR	4/5 (80%) HL, 1/5 (20%) unchanged.	3/5 (60%) required labyrinthectomy to control vertigo.	Ariagno (34)
Fick Sacculotomy	NR	1–10 years.	3/4 (75%)	NR	NR	4/4 (100%) complete HL after operation.	1/4 (25%) had recovery then return of vertigo, underwent labyrinthectomy.	Pavla et al. (35)
Fick Sacculotomy	Middle-aged MD diagnosis.	Mean 17 years.	14/17 (82%) (Class A–C)	NR	NR	HL > 15 dB in 11/17 (64%), 3/17 (18%) had complete HL. 2/17 (12%) had hearing gain >15 dB.	3/17 (18 %) labyrinthectomy due to incomplete vertigo control.	Weilinga and Smyth (36)
Cody Tack procedure	Mean age 50, 52% female. Symptom duration 8 months−7 years.	33% 6–24 months, 67% > 12 months.	38/42 (91%) with > 75% improvement in vertigo frequency.	29/35 (82%)	19/41 (46%)	6/42 (14%) experienced > 20 dB HL, 7/42 (17%) experienced >20 dB hearing improvement. Traumatic cases associated with HL.	2/42 (5%) experienced recurrent vertigo following improvement. Mild postural unsteadiness post-op was common.	Cody et al. (37)
Cody Tack procedure	Unilateral MD	3–6 months.	16/20 (80%)	15/20 (75%)	10/20 (50%), worsened in 2/20 (10%).	2/20 (10%) improved hearing, 7/20 (35%) worsened, 4/20 (20%) complete HL.	3/20 (15%) underwent labyrinthectomy. One experienced large crack in stapes footplate with perilymph leak and hearing loss.	Shea and Cottrell (38)
Cody Tack procedure	67/91 (74%) had > 1 vertigo attack per week, 75/91 (82%) had > 50 dB HL.	1–44 months.	85/91 (93%) with >75% reduction, 64/91 (70%) had no vertigo.	NR	NR	11/91 (12%) experienced air conduction loss >20 dB.	5/91 (5%) patients had improved vertigo after revisional operation. 6/91 (7%) severe vertigo returned after initial recovery.	Cody (39)
Cody Tack procedure	NR	> 6 months.	23/45 (51%)	NR	NR	6/45 (13%) with hearing improvement, 20/45 (44%) with HL.	12/45 (27%) underwent labyrinthectomy.	Pulec (28)
Cody Tack procedure	Unilateral MD, unresponsive to medical treatment, severe symptoms (>1 disabling attack/week), desire to avoid labyrinthectomy.	1–3 years.	12/13 (92%) experienced improvement >75%.	9/13 (69%)	8/13 (62%)	3/13 (23%) had >20 dB PTA decrease, 6/13 (46%) had >20 dB SRT decrease. 0/13 (0%) had improvement.	1/13 (8%) underwent labyrinthectomy.	Burgert et al. (40)
Cody Tack procedure	Mean 45 years old. Unsuccessful prior drug therapy.	NR	7/25 (28%) improvement, 17/25 (68%) experienced no change.	NR	NR	17/25 (68%) worsened hearing (> 10 dB), 2/25 (8%) had >10 dB gain.	12/25 (48%) underwent labyrinthectomy.	Jennings et al. (41)
Cody Tack procedure	Middle-aged.	Mean 12 years.	17/19 (89%)	NR	NR	10/19 (53%) had >15 dB HL, 3/19 (16%) experienced complete HL.	1/19 (5%) had cracked footplate with perilymph leak. 2/19 (11%) underwent subsequent labyrinthectomy.	Weilinga and Smyth (36)
Cochleo-sacculotomy	Patients selected for severity: 1–10 vertigo attacks per month.	1–24 months.	45/51 (88%). Three revision procedures included as improvement.	NR	NR	12/51 (23%) had >20 dB PTA loss, 35/51 (68%) had > 20 dB loss at 8 kHz.	1/51 (2%) postoperative otitis media. 6/51 (12%) had vertigo recurrence after improvement.	Schuknecht (26)
Cochleo-sacculotomy	NR	Mean 6 months.	9/14 (64%) fully relieved, 3/14 (21%) improved.	NR	NR	11/14 (79%) experienced severe HL (> 15 dB PTA) in weeks after procedure. 7/14 (50%) experienced HL at 6 months.	8/14 (57%) patients experienced acute unsteadiness. 6/14 (43%) showed unilateral decrease in vestibular function.	Silverstein et al. (42)
Cochleo-sacculotomy	Unilateral MD, > 50 dB HL, unsuccessful medical treatment.	> 12 months.	9/9 (100%)	NR	0/9 (0%)	6/9 (67%) severe HL. 3/9 (33%) low frequency hearing improvement but high frequency HL.	1/9 (11%) experienced positional vertigo post-operatively for 2 weeks.	Dionne (43)
Cochleo-sacculotomy	NR	2–3 years.	19/23 (83%) permanently relieved (3/23 (13%) after repeat procedure.)	NR	NR	2/23 (9%) experienced complete HL	Sense of disequilibrium or giddiness in first postoperative week. 2/23 (9%) underwent labyrinthectomy.	Montandon et al. (44)
Cochleo-sacculotomy	Average age 54 years, unsuccessful prior drug therapy.	NR	14/21 (67%)	NR	NR	10/21 (48%) had >10 dB HL, 5/21 (24%) had complete HL.	3/21 (14%) required a second procedure to control vertigo	Jennings et al. (41)
Cochleo-sacculotomy	Mean age 72.8 years.	6–38 months (mean 17.4).	2/11 (18%) fully relieved, 5/11 (45%) improved. Total 7/11 (64%)	NR	NR	Statistically significant decrease in PTA, SRT, SD among cohort.	4/11 (36%) required second procedure for vertigo control. All patients tolerated procedure well.	Giddings et al. (45)
Cochleo-sacculotomy	Mean age 40 years, 48% female, all non-smokers. 9/23 (39%) severe HL, 14/23 (61%) profound HL	NR	22/23 (96%)	NR	No change in tinnitus−0/15 (0%)	No hearing recovery, 9/23 (39%) worsening to profound HL.	NR	Sohielipour et al. (46)
Otic-perotic shunt	MD patients with poor baseline hearing.	NR	16/21 (76%)	NR	NR	5/21 (24%) had significant HL, 2/21 (10%) experienced complete HL.	Mild acute unsteadiness following procedure. 4/21 (19%) required labyrinthectomy.	Pulec (28, 29)
Cryosurgical otic-perotic shunt	Medical treatment ineffective, >40 dB HL at baseline.	6 months −1 year. 11/80 (14%) lost to follow-up.	48/69 (70%) with no HL > 20 dB and relieved of vertigo	NR	“Relieved or improved in about half”	3/69 (4%) had HL > 20 dB, may have been associated with perilymph leak.	8/69 (10%) had temporary facial paralysis during cooling. 7/69 (9%) had tympanic membrane perforation.	House (47)
Intracochlear shunt	NR	NR	9/9 (100%)	9/9 (100%)	9/9 (100%)	8/9 (89%) returned to baseline hearing level, 1/9 (11%) complete HL.	NR	Shea (48)
Lateral canal plugging	MD with functional scales 5 or 6 (severe disability), mean age 48 years (range 22–75) 1.2:1 M:F.	16/28 (57%) > 2 years, 12/28 (43%) > 6 months.	25/28 (89%) for 6 months, 12/16 (75%) Class A or B at 2 years	NR	NR	5/28 (18%) postoperative deafness	3/28 (11%) underwent ablative procedure. 2/28 (7%) unilateral labyrinthitis.	Charpiot et al. (49)
Triple canal plugging	Unilateral MD, previously treated with sac decompression or shunt, ages 46-55.	2–5 years follow-up.	3/3 (100%) two Class A, one Class B.	3/3 (100%)	3/3 (100%)	3/3 (100%) had no changes > 10 dB.	Dizziness and slight vertigo up to 3 days after procedure.	Yin et al. (50)
Triple canal plugging	37 male, 42 Female, ages 29–68 (mean 52).	> 2 years.	78/79 (98.7%) Class A or B.	NR	NR	23/79 (29%) had > 10 dB HL.	NR	Zhang et al. (51)
Triple canal plugging	Severely symptomatic despite medical treatment, ages 45–61, two male, one female.	> 2 years.	3/3 (100%) two Class A, one Class B.	NR	NR	1/3 (33%) > 30 db HL, 2/3 (67%) no change.	NR	Gill et al. (52)

*NR, not reported; HL, hearing loss; MD, Meniere's disease; PTA, pure tone audiogram; SRT, speech recognition threshold; SD, speech discrimination*.

## Decompression of the Saccule: Fick'S Sacculotomy

The saccule is more commonly affected by endolymphatic hydrops in Meniere's disease than the semicircular canals, and the hydropic saccule can adhere to the stapes footplate (4, 53). As otologic surgeons became comfortable performing stapes surgery in the 1950s and 1960s, the surgical approach to the saccule became feasible. In 1964 Fick proposed a transcanal approach to generate a conduit in the saccule to relieve excess endolymph (30). He inserted a fine needle through a fenestration made in the stapes footplate to puncture the dilated hydropic saccule (Figure 1B). Fick reports that rupture of the saccule resulted in immediate decreases in the sensation of aural fullness, but often elicited vertigo and temporary hearing loss lasting up to one week. Early reports of long-term results following the sacculotomy seemed promising. In 60 patients with several years of follow-up, Fick reported a high rate of relief from vertigo, improvements in hearing, and reductions in tinnitus and aural fullness, although nearly a quarter of patients were lost to follow up (Table 1) (30, 31). Subsequent reports, however, noted more modest improvements in vertigo, high rates of worsened hearing and cases in which symptoms later recurred (32–35, 37). Notably, sacculotomy procedures in animal models of endolymphatic hydrops failed to improve hydrops, and significant atrophy of cochlear sensory, neural, and supporting cells was present, observations that could underlie the hearing loss reported by many patients (54). In a long-term follow-up study of patients undergoing Fick's sacculotomy, >80% retained adequate control of vertigo episodes, but 64% had hearing worsened from baseline (36). The location of the membranous wall of the saccule could not have been known during the surgery, and variability in the degree of saccular distension could account for some variability in surgical outcomes (47, 54). Together, publications suggested that the Fick sacculotomy provided relief from episodes of vertigo, but that relief could be temporary, was often accompanied by substantial hearing loss, and sometimes required repeat procedures or a subsequent ablative surgery to control vertigo. Nevertheless, Fick's procedure inspired many similar innovations.

## Repeated and Permanent Fistula Through the Cody Tack Procedure and Cochleosacculotomy

A hypothesis for the incomplete long-term benefit following sacculotomy was that the membranous labyrinth of the saccule would heal, or the stapes fenestra would close, potentially resulting in recurrent endolymphatic hydrops and recurrent symptoms of Meniere's disease. Therefore, several procedures were developed with the goals of providing a permanent fistula for endolymph clearance and sustained prevention of vertigo.

In one procedure Cody and colleagues placed a sharp tack through the stapes footplate and directed it toward the saccule (37, 39). Like the Fick sacculotomy, a distended saccule at time of operation should rupture by placing the tack (Figure 1C). Cody hypothesized that by leaving the tack in place, the saccule would leak endolymph repeatedly whenever excess volume accumulated, theoretically providing sustained relief of symptoms. Early results in 42 patients revealed that in the weeks following placement of the tack patients experienced improved aural fullness and fewer vertigo episodes, but variable effects on hearing and tinnitus (Table 1) (37). Vestibular function—presumably assessed by caloric testing—was unchanged or improved in 39 of 40 patients. The authors deduced that vertigo relief was therefore not due to ablation of vestibular function. A subsequent study with 49 additional patients and longer follow-up (54), as well as reports from other surgeons (38, 40), suggested that more experience with the procedure was associated with better auditory outcomes. The incidence of hearing loss after the operation, however, remained high (41). Long-term outcomes of the Cody tack procedure were excellent with respect to control of vertigo episodes, with 89% satisfied enough with vertigo control to avoid subsequent labyrinthectomy (36). Shorter tack length was associated with recurrent vertigo episodes, supporting the authors' hypothesis for how the tack might work (39). Other groups reported poorer outcomes for control of vertigo episodes, with more patients subsequently pursuing labyrinthectomy (41). In a long-term follow-up study that compared the tack procedure to Fick's sacculotomy, a greater proportion of patients that underwent the tack procedure maintained good hearing [47% class A or B by American Academy of Ophthalmology and Otolaryngology classification vs. 18%, (36)]. A hypothesis for this difference is not mentioned by the authors.

Schuknecht promoted an alternative approach to maintain decompression of the hydropic ear that he called cochleosacculotomy (26). A right-angled pick is inserted through the round window and advanced to fracture the basal osseous spiral lamina to form a fistula, followed by rupture of both the cochlear duct and a dilated saccule (Figure 1D). The goal was to create a permanent fistula between perilymph and endolymph compartments. Animal studies suggested that surgically creating a fistula through the basal osseous spiral lamina caused degeneration of cells near the surgery and impaired auditory responses for high frequency sound. Auditory responses to frequencies distal to the lesion site, however, remained unaffected (55, 56). Histological analysis revealed that about half of the animals retained a fistula. In the initial characterization of patients with Meniere's disease who underwent cochleosacculotomy, success rates were high for control of vertigo episodes (Table 1), but some experienced recurrent vertigo episodes, and many lost hearing (26).

Later studies of cochleosacculotomy performed by other surgeons and across different patient age groups revealed largely consistent success rates for the control of vertigo episodes (Table 1). Severe sensorineural hearing loss was common in the first weeks after surgery, and while modest recovery in hearing was observed with longer follow-up, impaired hearing often persisted more than one year after the procedure (27, 41–43, 46). Unlike the findings in animals, however, hearing loss was not limited to frequencies sensed near the lesion, suggesting that injury to the cochlea in patients was more diffuse than originally anticipated (27). In addition, half of patients had general unsteadiness and vestibular impairment one-month after surgery (42). While the surgery was extremely well-tolerated by elderly individuals, long-term relief from vertigo episodes was poorer than expected in older patients, and hearing loss was experienced at a greater rate (45). In a comparative translational study, cochleosacculotomy failed to resolve endolymphatic hydrops or create a permanent fistula in an animal model of endolymphatic hydrops despite the improvement of symptoms reported by the cohort of patients. The authors suggested that successful outcomes in patients following surgical procedures on the labyrinth may be due to non-specific reactive changes in the ear with endolymphatic hydrops (44) or by “mini-labyrinthectomy” causing impaired vestibular function that had been observed in some, but not all, patients (42, 43).

## Shunts Within the Cochlea: Intracochlear Shunt and Otic-Perotic Shunt

Also intending to create a permanent connection between endolymph and perilymph, John Shea, Jr. developed the intracochlear shunt procedure for Meniere's disease in the 1980s (48, 57). Shea had initially proposed a procedure called sacculocentesis, in which the stapes footplate is removed, and a micropipette is used to extract endolymph from the saccule, without mixing endolymph and perilymph. Most patients (87%) experienced relief from vertigo episodes, but hearing worsened in one-third of patients after 2 years follow up, and he abandoned the procedure (48). In the intracochlear shunt procedure, Shea created a hole in the cochlea between the oval and round windows, opening into the scala tympani. Using a pick, a puncture was then made in the basilar membrane allowing endolymph of the scala media to communicate with perilymph of the scala tympani. According to Shea, patients undergoing this procedure experienced immediate unsteadiness and decreased hearing but reported improved aural fullness and tinnitus as well as relief from episodic vertigo. The hearing loss improved over time, with 8 of 9 patients returning to baseline hearing (Table 1) (48). In an animal model of endolymphatic hydrops, creating a fistula using Shea's intracochlear shunt procedure led to less distention of Reissner's membrane one week after the procedure, with only mild degenerative changes in the adjacent region of the organ of Corti (58). One month after the procedure, however, extensive hydrops was present in all animals along with severe degeneration of the organ of Corti and the macula of the saccule, suggesting that creating a fistula in the basilar membrane between endolymph and perilymph is insufficient to reduce endolymphatic hydrops permanently.

To create a permanent shunt between endolymph and perilymph in the cochlea, Pulec—crediting William House—placed a platinum tube through the basilar membrane at the base of the cochlea using only local anesthesia. The tube was loaded on a right-angle pick, introduced through the round window and left in place. Like prior procedures, the aim was to enable pressure equalization and mixing between the endolymph of the scala media and perilymph of the scala tympani (Figure 1E) (28, 29). In another attempt to create an otic-perotic shunt, House placed a cryosurgical probe on thinned bone over the promontory of the cochlea. The goal was to destroy tissue of the membranous labyrinth at the saccule and basal cochlear duct, presumably causing a connection between endolymph and perilymph spaces, but without surgically opening into the perilymph space (47). Like other manipulations of the membranous labyrinth, both mechanical and cryosurgical shunt formation relieved patients of episodic vertigo (Table 1). However, a severe hearing loss often accompanied the tube placement in the basilar membrane, and patients undergoing cryosurgery had mixed results and other complications including temporary facial paresis (10%) and tympanic membrane perforations (9%). Given the challenge of blindly implanting a tube into the cochlear duct and the challenges associated with implementing cryosurgery, these procedures were not widely adopted.

## Semicircular Canal Plugging

More recently, surgeons began opening and plugging the semicircular canals in patients with Meniere's disease, drawing lessons from similar procedures performed for the treatment of superior semicircular canal dehiscence syndrome and refractory benign paroxysmal positional vertigo of the posterior semicircular canal. For these conditions, surgeons occlude a semicircular canal to block aberrant stimulation of that canal, while intending to preserve function of the other inner ear structures. Lateral semicircular canal plugging in patients with Meniere's disease is performed with the goal that targeted ablation of endolymph movement in the lateral canal could address hypothesized intermittent high pressure during hydrops and control symptoms of vertigo. A 2 mm fenestra is opened in the bony canal wall, and a plug composed of fibrinogen glue and temporalis fascia is inserted through the fenestra to compress the membranous labyrinth and prevent endolymph movement (49). Prospective studies suggest that long-term canal paresis controls episodic vertigo (~75%, Table 1) (49). Plugging of all semicircular canals on the affected side has also been performed for patients with Meniere's disease (50–52), achieving similar levels of vertigo control to lateral canal occlusion (Table 1). A larger retrospective study comparing occlusion of all three semicircular canals to endolymphatic sac decompression suggested higher rates of vertigo control with canal occlusion, but also greater hearing loss (58).

## Conclusions and Future Considerations

The history of Meniere's disease is rich with surgical innovation. Aside from the frequent association between patients with symptoms of Meniere's disease and endolymphatic hydrops on histology, however, the etiology remains a mystery, making targeted medical treatments impossible. Furthermore, the role that endolymphatic hydrops plays in the disease is uncertain. Nevertheless, surgeons hypothesized that endolymphatic hydrops causes the symptoms of Meniere's disease and have attempted to intervene on this finding. The history of these surgical interventions has paralleled technological developments within otology. Lateral semicircular canal fenestration, the introduction of the operating microscope, the development of stapes surgery, and now increasing experience with semicircular canal plugging have each influenced procedures that aim to address the finding of endolymphatic hydrops. Whether the procedures improved endolymphatic hydrops was unknown, since historically this could be assessed only by histology on post-mortem specimens. The broad goals of these procedures are eliminating episodic vertigo while preserving hearing. Assessing the success of interventions is difficult due to the natural history of Meniere's disease in which cessation of vertigo episodes over time is common (59–61). While hearing loss remains frequent following these procedures, many individuals who underwent procedures experienced relief from vertigo without hearing loss. While this relief might have occurred despite the intervention, other variables such as technical differences among surgeons and other patient-specific factors like extent of hydrops (54) may underly undesirable complications and variable outcomes. Pulec commented in 1969: “one might speculate that if this operation could be accomplished in a more delicate, precise fashion, the desired results in every case could be obtained” (29). We continue to learn about operating on the membranous labyrinth, now from surgeries for semicircular canal dehiscence (62) and vestibular implants (63). Inevitably, advances in surgical technology including pre-operative imaging, operative robotics and improved magnification will lead to new surgical approaches on the membranous labyrinth. Hopefully, this also will improve outcomes for patients with inner ear disorders like Meniere's disease. Our field, however, must understand better how endolymphatic hydrops relates to the symptoms of Meniere's disease.

## Author Contributions

Conceptualization: BW; Investigation: CK and BW; Writing: CK and BW. All authors listed have made a substantial, direct, and intellectual contribution to the work and approved it for publication.

## Funding

CK is supported by a National Research Service Award F30DC018711 from the National Institutes of Health. BW is supported by clinician-scientist award K23DC018302 from the National Institutes of Health and an emerging research grant on Meniere's disease by the Hearing Health Foundation.

## Conflict of Interest

The authors declare that the research was conducted in the absence of any commercial or financial relationships that could be construed as a potential conflict of interest.

## Publisher's Note

All claims expressed in this article are solely those of the authors and do not necessarily represent those of their affiliated organizations, or those of the publisher, the editors and the reviewers. Any product that may be evaluated in this article, or claim that may be made by its manufacturer, is not guaranteed or endorsed by the publisher.

## References

[1] Baloh RW. Prosper Meniere and his disease. Arch Neurol. (2001) 58:1151–6. 10.1001/archneur.58.7.1151 11448308

[2] Hallpike CS Cairns H. Observations on the pathology of Meniere's syndrome. J Laryngol Otol. (1938) 63:625–55. 10.1017/S0022215100003947 19991672 PMC2076781

[3] Yamakawa K. Uber die pathologisch Veranderung bei einem Ménière-Kranken. J Otorhinolaryngol Soc Jpn. (1938) 4:2310–2.

[4] Sperling NM Paparella MM Yoon TH Zelterman D. Symptomatic versus asymptomatic endolymphatic hydrops: a histopathological comparison. Laryngoscope. (1993) 103:277–85. 10.1288/00005537-199303000-00007 8441315

[5] Fraysse BG Alonso A House WF. Meniere's disease and endolymphatic hydrops: clinical-histopathological correlations. Ann Otol Rhinol Laryngol. (1980) 89:1–22. 10.1177/00034894800896S201 6779694

[6] Schuknecht HF Gulya AJ. Endolymphatic hydrops: an overview and classification. Ann Otol Rhinol Laryngol. (1983) 92:1–20. 10.1177/00034894830920S501 6414357

[7] Merchant SM Adams JC Nadol JB Jr. Pathophysiology of Meniere's syndrome: are symptoms caused by endolymphatic hydrops? Otol Neurotol. (2005) 26:74–81. 10.1097/00129492-200501000-00013 15699723

[8] Rauch SD Merchant SN Thedinger BA. Meniere's syndrome and endolymphatic hydrops: Double-blind temporal bone study. Ann Otol Rhinol Laryngol. (1989) 98:873–83. 10.1177/000348948909801108 2817679

[9] Liston SL Paparella MM Mancini F Anderson JH. Otosclerosis and endolymphatic hydrops. Laryngoscope. (1984) 94:1003–7. 10.1288/00005537-198408000-00001 6748826

[10] Sharon JD Trevino C Schubert MC Carey JP. Treatment of Meniere's disease. Curr Treat Options Neurol. (2015) 17:14. 10.1007/s11940-015-0341-x 25749846

[11] Knapp HJ. A Clinical Analysis of the Inflammatory Affections of the Inner Ear. New York: W Wood & Company (1871).

[12] Burnett CH. The relief of chronic deafness, tinnitus aurium and tympanic vertigo, by removal of the incus and stapes. Read in the section on laryngology and otology at the forty-fourth annual meeting of the American Medical Association. J Am Med Assoc. (1893) 21:760–3. 10.1001/jama.1893.02420730010002a25996397

[13] Burnett CH. Chronic ear-vertigo (Meniere's Disease); it's mechanism and surgical treatment. Philadelphia Med J. (1900) 6:554–7.

[14] Jenkins GJ. Labyrinthine vertigo (Menière's symptoms—non-infective) treated by operation. Proc R Soc Med. (1911) 4:116–20. 10.1177/003591571100401038 PMC2004374 19975356

[15] Lake R. Ten cases of operation for Ménière's disease (aural vertigo). Lancet. (1911) 177:1569–70. 10.1016/S0140-6736(00)78274-2

[16] Portmann G. The saccus endolymphaticus and an operation for draining for the relief of vertigo. Proc R Soc Med. (1927) 20:1862–7. 10.1177/003591572702001238 19986124 PMC2101476

[17] House WF. Subarachnoid shunt for drainage of endolymphatic hydrops: a preliminary report. Laryngoscope. (1962) 72:713–29. 10.1288/00005537-196206000-00003 14449308

[18] Sood AJ Lambert PR Nguyen SA Meyer T. A endolymphatic sac surgery for Meniere's disease: a systematic review and meta-analysis. Otol Neurotol. (2014) 35:1033–45. 10.1097/MAO.0000000000000324 24751747

[19] Kimura RS. Experimental blockage of the endolymphatic duct and sac and its effect on the inner ear of the guinea pig. A study on endolymphatic hydrops. Ann Otol Rhinol Laryngol. (1967) 76:664–87. 10.1177/000348946707600311 6046009

[20] Saliba I Gabra N Alzahrani M Berbiche D. Endolymphatic duct blockage: a randomized controlled trial of a novel surgical technique for Meniere's disease treatment. Otolaryngol Head Neck Surg. (2015) 152:122–9. 10.1177/0194599814555840 25403881

[21] Gluth MB. On the relationship between Meniere's disease and endolymphatic hydrops. Otol Neurotol. (2020) 41:242–9. 10.1097/MAO.0000000000002502 31746815

[22] Day KM. Labyrinth surgery for Meniere's disease. Laryngoscope. (1943) 53:617–30. 10.1288/00005537-194310000-00001 13720340

[23] Lindsay JR. Labyrinthine surgery for Meniere's disease. Laryngoscope. (1949) 59:22–34. 10.1288/00005537-194901000-00003 18123734

[24] Cawthorne T. Otosclerosis. J Laryngol Otol. (1955) 69:437–56. 10.1017/S0022215100050933 13242969

[25] Femenic B. Drainage operation in cases of the hydrops of the labyrinth and its influence on hearing. J Laryngol Otol. (1961) 75:640–6. 10.1017/S0022215100058230 13698814

[26] Schuknecht HF. Cochleosacculotomy for Meniere's disease: theory, technique, and results. Laryngoscope. (1982) 92:853–8. 10.1288/00005537-198208000-00004 7098734

[27] Kinney WC Nalepa N Hughes GB Kinney SE. Cochleosacculotomy for the treatment of Meniere's disease in the elderly patient. Laryngoscope. (1995) 105:934–7. 10.1288/00005537-199509000-00012 7666728

[28] Pulec JL. The surgical treatment of vertigo. Laryngoscope. (1969) 79:1783–822. 10.1288/00005537-196910000-00009 4899304

[29] Pulec JL. The otic-perotic shunt. Otolaryngol Clin North Am. (1968) 1:643–8. 10.1016/S0030-6665(20)33323-5

[30] Fick IAvN. Decompression of the labyrinth: A new surgical procedure for Meniere's disease. Arch Otolaryngol. (1964) 79:447–58. 10.1001/archotol.1964.00750030458005 14120668

[31] Fick IAvN. Meniere's disease: etiology and a new surgical approach: sacculotomy. J Laryngol Otol. (1966) 80:288–306. 10.1017/S0022215100065245 5907837

[32] Proud GO. The fick procedure. Arch Otolaryngol. (1965) 81:435. 10.1001/archotol.1965.0075005044602525996397

[33] Arslan M. Dr. Fick's operation. Arch Otolaryngol. (1965) 81:435–7. 10.1001/archotol.1965.0075005044602625996397

[34] Ariagno RP. Surgical dilemma in Meniere's disease. Arch Otolaryngol. (1966) 83:320–3. 10.1001/archotol.1966.00760020322005 5907022

[35] Pavla T Karja J Pavla A. Surgical treatment of Meniere's disease. Acta Otol Laryngol. (1976) 82:303–7. 10.3109/00016487609120912 983695

[36] Wielinga EWJ Smyth GDL. Long-term results of sacculotomy in older patients. Ann Otol Rhinol Laryngol. (1989) 98:803–6. 10.1177/000348948909801010 2802463

[37] Cody DTR Simonton KM Hallberg OE. Automatic repetitive decompression of the saccule in endolymphatic hydrops (Tack operation) Preliminary report. Laryngoscope. (1967) 77:1480–501. 10.1288/00005537-196708000-00017 6034864

[38] Shea JJ Cottrell RE. The cody tack sacculotomy for endolymphatic hydrops. J Tenn Med Assoc. (1968) 61:889–90. 5678496

[39] Cody DTR. The tack operation for endolymphatic hydrops. Laryngoscope. (1969) 79:1737–44. 10.1288/00005537-196910000-00005 5345407

[40] Burgert P Goode RL Simmons FB Smith MFW. The cody tack operation – an evaluation. Laryngoscope. (1972) 82:2169–73. 10.1288/00005537-197212000-00004 4648365

[41] Jennings RP Reams CL Jacobson J Cole JM. Results of surgical treatment for Meniere's disease. Otolaryngol. Head Neck Surg. (1989) 100:195–99. 10.1177/019459988910000304 2496379

[42] Silverstein H Hyman S Silverstein D. Cochleosacculotomy. Otolaryngol. Head Neck Surg. (1984) 92:63–6. 10.1177/019459988409200114 6422418

[43] Dionne J. Cochleosacculotomy. J Otolaryngol. (1985) 14:59–61. 4068093

[44] Montandon PB Hausler RJ Kimura RS. Treatment of endolymphatic hydrops with cochleosacculotomy. Otolaryngol Head Neck Surg. (1985) 93:615–21. 10.1177/019459988509300509 3932929

[45] Giddings NA Shelton C O'Leary MJ Brackmann DE. Cochleosacculotomy revisited Long-term results poorer than expected. Arch Otolaryngol Head Neck Surg. (1991) 117:1150–2. 10.1001/archotol.1991.01870220098017 1910702

[46] Sohielipour S Abtahi SH Soltani M Khodadadi H. Comparison the results of two different vestibular system surgery in patients with persistent Meniere's disease. Adv Biomed Res. (2015) 4:198. 10.4103/2277-9175.166134 26601086 PMC4620615

[47] House WF. Cryosurgical treatment of Meniere's disease. Arch Otolaryngol. (1966) 84:616–29. 10.1001/archotol.1966.00760030618005 5957137

[48] Shea JJ. Intracochlear shunt. Otolaryngol Clin North Am. (1983) 16:293–9. 10.1016/S0030-6665(20)32934-0 6856309

[49] Charpiot A Rohmer D Gentine A. Lateral semicircular canal plugging in severe Meniere's disease: a clinical prospective study about 28 patients. Otol Neurotol. (2010) 31:237–40. 10.1097/MAO.0b013e3181ca85a2 20101162

[50] Yin S Chen Z Yu D Wu Y Shi H Zhou H. Triple semicircular canal occlusion for the treatment of Meniere's disease. Acta Otolaryngol. (2008) 128:739–43. 10.1080/00016480701730000 18568514

[51] Zhang D Fan Z Han Y Lv Y Li Y Wang H. (2016) Triple semicircular canal plugging: a novel modality for the treatment of intractable Meniere's disease, *Acta Oto Laryngol*. (2016) 136:1230–5. 10.1080/00016489.2016.1206966 27434132

[52] Gill C Muzaffar J Kumar R Irving R. Triple canal occlusion for the treatment of intractable Meniere's disease. Otol Neurotol. (2021) 42:116–20. 10.1097/MAO.0000000000002841 33201079

[53] Schuknecht HF. The pathophysiology of Meniere's disease. Am J Otol. (1984) 5:526–7. 6393773

[54] Kimura RS Schuknecht HF Ota CY Jones DD. Experimental study of sacculotomy in endolymophatic hydrops. Arch Otol Rhino Laryng. (1977) 217:123–37. 10.1007/BF00665532 578728

[55] Schuknecht HF Sutton S. Hearing losses after experimental lesions in the basal coil of cochlea. Arch Otolaryngol. (1953) 57:129–42. 10.1001/archotol.1953.00710030148002 13007264

[56] Schuknecht HF Neff WD. Hearing losses after apical lesions in the cochlea. Acta Otolaryngol. (1952) 42:263–74. 10.3109/00016485209120353 12976099

[57] Shea JJ Yoo TJ O'Connor AF Tomoda K Orchik DJ. Recents advances in Meniere's disease research in the United States. Equilib Res. (1983) 42:86–100. 10.3757/jser.42.86

[58] Yazawa Y Kitano H Suzuki M Kitanishi T Kitajima KE. Effects of endolymphatic-perilymphatic fistula on endolymphatic hydrops in guinea pig. Otolaryngol Head Neck Surg. (2000) 122:119–23. 10.1016/S0194-5998(00)70159-8 10629498

[59] Silverstein H Smouha E Jones R. Natural history vs. surgery for Meniere's disease. Otolaryngol Head Neck Surg. (1989) 100:6–16. 10.1177/019459988910000102 2493618

[60] Thomsen J Tos M Johnsen NJ. Placebo effect in surgery for Meniere's disease. Arch Otolaryngol. (1981) 107:271–7. 10.1001/archotol.1981.00790410009002 7013741

[61] Welling DB Nagaraja HN. Endolymphatic mastoid shunt: a reevaluation of efficacy. Otolaryngol Head Neck Surg. (2000) 122:340–345. 10.1067/mhn.2000.101575 10699806

[62] Beyea JA Agrawal SK Parnes LS. Transmastoid semicircular canal occlusion: a safe and highly effective treatment for benign paroxysmal positional vertigo and superior canal dehiscence. Laryngoscope. (2012) 122:1862–6. 10.1002/lary.23390 22753296

[63] Chow MR Ayiotis AI Schoo DP Gimmon Y Lane KE Morris BJ. Posture, gait, quality of life, and hearing with a vestibular implant. N Engl J Med. (2021) 384:521–32. 10.1056/NEJMoa2020457 33567192 PMC8477665

